# Tailoring mechanical and thermal behavior of HDPE via Ba–Sr–Ca hexaferrite incorporation

**DOI:** 10.1038/s41598-026-57097-x

**Published:** 2026-06-18

**Authors:** Mohamed S. Badawi, K. Habanjar, Mohammad Ali Shaib, M. Y. El Sayed, Amro Obeid, Ramy. M. Moussa, Mohamed. I. Badawi, R. Awad

**Affiliations:** 1 Faculty of Science, Alamein International University, Alamein City, Matrouh Governorate Egypt; 2 Department of Physics, Faculty of Science, Alexandria University, Alexandria, Egypt; 3 Department of Physics, Faculty of Science, Beirut Arab University, Beirut, Lebanon; 4 Public Health Department, Faculty of Health Sciences, Modern University for Business and Science, Beirut, Lebanon; 5 Lebanese Atomic Energy Commission, National Council for Scientific Research, Beirut, Lebanon; 6 Department of Physics, School of Sciences and Engineering, American University in Cairo (AUC), AUC Avenue, P.O. Box 7, New Cairo, 11835 Egypt; 7 Department of Biomedical Equipment Technology, Faculty of Applied Health Sciences Technology, Pharos University in Alexandria, Alexandria, Egypt

**Keywords:** BaSrCa, HDPE, Young’s modulus, Activation energy, thermal stability, Engineering, Materials science, Nanoscience and technology

## Abstract

Using mechanical ball milling at filler loadings of 5–20 wt%, high-density polyethylene (HDPE) nanocomposites reinforced with BaSrCa hexaferrite nanoparticles were created, and their structural, mechanical, and thermal performance was thoroughly examined. The M-type hexaferrite phase was successfully incorporated into the HDPE matrix, as demonstrated by X-ray diffraction, and the composite crystallite size gradually decreased from 59.31 nm (5 wt%) to 50.38 nm (20 wt%). Effective filler integration was confirmed by Rietveld refinement, which showed a gradual increase in the BaSrCa volume percentage from 4.89% to 19.25% while the HDPE fraction fell proportionally. HDPE lattice characteristics (a: 7.418 → 7.410 Å, b: 4.940 → 4.933 Å, and c: 2.485 → 2.475 Å) show slight contractions that suggest interfacial stress and limited polymer chain mobility. As the filler content increased, mechanical testing showed a noticeable stiffening impact. Young’s modulus rose from 144.35 MPa for pure HDPE to 191.35 MPa at 20 wt% BaSrCa (5 mm/min) and 239.04 MPa at 20 mm/min. While yield stress increased significantly from 16.48 MPa to 25.37 MPa, ultimate tensile strength showed a slight improvement from 24.44 MPa to 27.33 MPa. On the other hand, strain at break dropped dramatically from 536% to about 70%, indicating lower ductility because of filler-induced constraint. At greater BaSrCa concentrations, the thermal study revealed improved stability with a delayed commencement of deterioration and a higher residual mass. Overall, the findings show that BaSrCa nanoferrites considerably improve HDPE’s strength, stiffness, and thermal stability at the expense of ductility. These composites are promising options for lightweight structural, electromagnetic, and heat-resistant applications because an ideal filler range of 10–15 wt% is found, providing a balanced combination of mechanical reinforcement and structural integrity.

## Introduction

High-density polyethylene (HDPE) is a semi-crystalline polymer material that is used frequently, especially in bottles, geomembranes, pipes, and packaging. This is due to its low weight, strength, wear resistance, impact resistance, durability, and resistance to chemicals^1^. Despite its many benefits, HDPE has limited barrier properties, including low thermal and electrical conductivity, and no electromagnetic (EM) shielding capability^2^. These shortcomings can be mitigated by modifying HDPE properties via different techniques. One of the easiest ways is to add nanoparticles as a filler in the form of polymer nanocomposites^3^.

Polymer nanocomposites reinforced with nanoparticles exhibit enhanced properties due to synergistic effects^4^. By incorporating nanoparticles, the electrical, optical, thermal, and mechanical characteristics can be adjusted. Also, this method maintains the lightweight and processable nature of the polymer. Nanotechnology has led to the development of various functional nanoparticles to reinforce polymer materials^5^. This sets the stage towards multifunctional and high-performance polymers^6^.

Ferrite-based nanofillers are particularly important to provide electromagnetic and magnetic behavior to polymers^7^. Of these, M-type hexaferrites, with the general chemical formula Me$\:{Fe}_{12}{O}_{19}$ (where Me = Ba, Sr, Ca), have a magnetoplumbite hexagonal structure, as compared to spinel ferrites^5^. These materials are characterized by their high Curie temperatures, strong uniaxial anisotropy, high hardness, chemical stability, and high electrical resistivity, which minimizes eddy current losses at high frequencies^8,9^. In detail, Ba_0.5_Sr_0.25_Ca_0.25_Fe_12_O_19_ (BaSrCa) draws together the most beneficial characteristics of Ba-, Sr-, and Ca-substituted hexaferrites. It is a potentially good material in the context of permanent magnets, microwave devices, recording heads, antenna rods, and transformer commercial components^10,11^.

In the present work, various loadings of BaSrCa nanoparticle (5, 7.5, 10, 15, and 20 wt%) were explored to assess the structural, morphological, mechanical, and thermal characteristics of HDPE-based nanocomposites. This has not been comprehensively reported in the literature. Hence, BaSrCa nanoparticles were synthesized via the co-precipitation method. These nanoparticles were then introduced into HDPE in different weights through the ball milling process to provide nanocomposites. The structural and morphological characteristics were determined with X-ray diffraction (XRD), transmission electron microscope (TEM), Fourier-transform infrared spectroscopy (FTIR), energy-dispersive X-ray spectroscopy (EDX), and scanning electron microscope (SEM). The analysis of mechanical properties was done via the tensile test. Thermal properties were evaluated by using thermogravimetric analysis (TGA) and differential scanning calorimetry (DSC). The intention is to find the optimum nanoparticle loading at which the multifunctionality of HDPE emerges, and the lightweight and cost-effective benefits will remain unchanged. Unlike previous studies, this work demonstrates how hexaferrite incorporation not only enhances strength and thermal stability but also introduces multifunctional capabilities relevant to electronics, telecommunications, and the reduction of electromagnetic pollution.

## Experimental and characterization techniques

The wet chemical co-precipitation technique was used to produce BaSrCa nanoparticles. 1 M of salt chloride using BaCl_2_·2H_2_O (98%), SrCl_2_·6H_2_O (99%), CaCl_2_ (97%), and FeCl_3_·6H_2_O (98%) were first weighed stoichiometrically. Different salt solutions were obtained when the salts were dissolved in deionized water, with continuous stirring at room temperature for 1 h. Next, BaCl_2_·2H_2_O, SrCl_2_·6H_2_O and CaCl_2_ solutions were combined with FeCl_3_·6H_2_O solution, via magnetic stirring. The resultant mixture of BaSrCa solution was drop-wise subjected to 3 M sodium hydroxide titration, respectively. After reaching a pH of 12, the salt solutions were heated at 80 °C for 2 h. Following, the resultant precipitates undertook extensive filtering and washing, using a mixture of solvents (50% ethanol and 50% deionized water), until the pH became neutral. Afterward, the precipitates were dried at 100 °C for 18 h. The resulting powders were then ground and calcined at 950 °C for 2 h.

This study begins with the synthesis and characterization of BaSrCa nanoparticles, produced via the co-precipitation method. Subsequently, nanocomposites based on BaSrCa-reinforced HDPE were prepared using mechanical ball milling with various weight percentages of the synthesized nanoparticles. The structural and morphological properties of the samples were examined using X-ray diffraction (XRD) and transmission electron microscopy (TEM).

A new high-density polyethylene (HDPE) nanocomposite system was thus developed by incorporating different loadings of the prepared hexaferrite nanoparticles through mechanical ball milling. The ball milling procedure was carried out using a planetary ball mill at a rotational speed of 450 rpm for a one-hour duration with intermittent pauses to prevent overheating. A ball-to-powder weight ratio of 10:1 was maintained to ensure effective milling. The milling was performed at room temperature in a dry medium using agate balls. After completion, the milled powder was collected and dried (if wet milling was used) before further characterization or use. The aim was to investigate the structural, morphological, mechanical, and thermal properties of the resulting nanocomposites and to determine the optimal filler percentage for producing a multi-functional, lightweight, and low-cost material. This approach combines the advantageous mechanical and thermal properties of HDPE with the magnetic and electromagnetic shielding characteristics of the hexaferrite nanoparticles, enabling potential applications in electronics, telecommunications, and electromagnetic pollution protection. Using the compression molding technique, which functioned at 170 °C and 50 rpm, BaSrCa/HDPE composites were fabricated by melting HDPE for 10 min firstly in a roll mixer machine (XK400). Then, adding 0, 5, 7.5, 10, 15, and 20 wt% of BaSrCa nanoparticles (average particle size: ~30 nm), and mixing for 15 min. The mixture was stirred to achieve homogeneity and uniformity. The resulting specimens were pulled out from the mixer after milling and then extended into a rectangular stainless steel mold of dimensions (25 × 25 × 0.3 cm3). By using a hot press of 20 MPa performed at 170 °C, the samples were hard-pressed for 15 min and cooled by a water system at a rate of 20 °C/min. The specimens were cut into the required size to perform the needed measurements.

X-ray powder diffraction (XRD) was employed to examine the structure of the generated samples using a Bruker D8 Advance device with Cu-Kα radiation (λ = 1.5406 Å). The diffraction patterns generated by the diffractometer using the Rietveld method were refined using the Material Analysis Using Diffraction (MAUD) application. Fourier transform infrared (FTIR) spectra in the wavenumber range of 4000–400 cm^−1^ were acquired using a Nicolet iS5 spectrometer with a resolution of 4 cm^−1^. The prepared composite samples were shaped into transparent pellets for measurement after being coarsely ground and mixed with spectroscopic-grade KBr. A Transmission Electron Microscope TEM (JOEL, JEM-2100) with an accelerating voltage of 20 kV was used to examine the microstructure, agglomeration, and particle size distribution. Additionally, the morphology and elemental content of the generated samples were examined using energy-dispersive X-ray spectroscopy (EDX) and scanning electron microscopy (SEM). These studies were conducted using a JEOL JCM-6000 PLUS with EX-54450U1S61 detector, which focused on different regions of the samples and had an operating voltage of 20 keV. To prevent the samples from charging, a thin layer of gold was sputter-coated onto them. An EDX attachment was used to verify the elemental composition. Mechanical tensile testing was conducted using the Tension/Compression Force Tester (Mark-10 Force Measurements), branded as ESM303. The samples were formed like dumbbells in compliance with the international standard ASTM-D638. Three specimens with identical weight percentages were used in the tensile test, which was carried out at three different speeds (5, 10, and 20 mm/min). A TGA-DTA/DSC SETARAM-Labsys was used to investigate the heat degradation of the composites. The chosen operational features included a heating rate of 10 °C/min in a pure nitrogen environment and a heating range of 25 °C to 600 °C. Samples weighing between 10 and 15 mg were placed in alumina (Al_2_O_3_) crucibles.

## Results and discussion

### XRD analysis

**Fig. 1:**
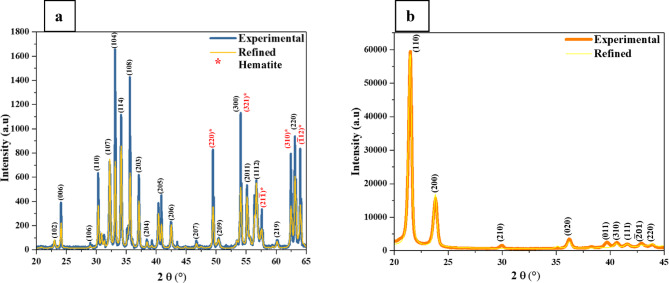
XRD patterns of **a** BaSrCa nanoparticle and **b** HDPE polymer.

Figure 1 displays the X-ray diffraction (XRD) pattern corresponding to the BaSrCa nanoparticles. All detected diffraction peaks were precisely indexed to the M-type hexaferrite crystalline structure, matching the P6_3_/mmc space group (JCPDS card no. 00-007-0276)^12^. This was verified through the Rietveld refinement executed via the MAUD software. Some traces of hematite (α-Fe_2_O_3_) were detected as a secondary impurity phase within the hexaferrite nanoparticles. This is a phenomenon that is recurrently documented in such systems^13^. This is rationalized by the partial reduction of Ba^2+^ and Sr^2+^ ions during thermal treatment, leading to the subsequent oxidation of residual Fe ions into Fe_2_O_3_^13^.

The extracted lattice parameters of HDPE and BaSrCa nanoparticles are recorded in Table 1. Also, the average crystallite size (D) was evaluated using the Debye-Scherrer equation as follows^14^:

1 $$\:D=k\lambda\:/\beta\:cos\theta\:$$

where $\:k$ denotes the Scherrer constant (0.9), *λ* represents the X-ray wavelength, *β* corresponds to the full width at half maximum (FWHM), and *θ* is the Bragg angle at the considered peak.

**Table 1:** Structural parameters for pure BaSrCa, pure HDPE, and α-Fe_2_O_3_ phases.

	Lattice parameters			D (nm)	Volume fraction (%)
	a (Å)	b (Å)	c (Å)		
BaSrCa	5.885	5.885	23.126	80.17	62.35
α-Fe_2_O_3_	5.032	5.032	13.75	–	37.66
HDPE	7.452	4.941	2.489	43.55	100

With lattice constants a = b = 5.885 Å and c = 23.126 Å, the BaSrCa hexaferrite has a hexagonal structure that is typical of M-type hexaferrites. It is confirmed to be the main magnetic phase in the composite by its moderate volume fraction (62.35%) and comparatively large crystallite size (D = 80 nm). With a volume fraction of 37.66%, the secondary phase, α-Fe_2_O_3_, exhibits reduced lattice characteristics. This suggests that there may be residual iron oxide or partial transformation during production, which could lead to more magnetic interactions inside the nanocomposite. On the other hand, HDPE has an orthorhombic crystalline structure with a crystallite size of around 43.5 nm and lattice parameters of a = 7.452 Å, b = 4.941 Å, and c = 2.489 Å. Since it serves as the continuous polymer matrix, its volume percentage is assumed to be 100%.

**Table 2:** Structural and refinement parameters of the HDPE/BaSrCa composites.

Sample	BaSrCa 5%	BaSrCa 7.5%	BaSrCa 10%	BaSrCa 15%	BaSrCa 20%
D (nm)	59.31	53.82	52.52	51.24	50.38
Volume fraction of BaSrCa (%)	4.89	6.74	10.50	14.75	19.25
Volume fraction of HDPE (%)	95.11	93.26	89.50	85.25	80.75
$\:\boldsymbol{a}$ (Å)	7.418	7.415	7.413	7.412	7.410
$\:\boldsymbol{b}$ (Å)	4.940	4.939	4.935	4.935	4.933
$\:\boldsymbol{c}$ (Å)	2.485	2.482	2.481	2.480	2.475

The structural characteristics of the HDPE/BaSrCa nanocomposites exhibit a predictable evolution with increased ferrite loading, according to the data in Table 2. As the ferrite concentration rises from 5% to 20%, the composite’s crystallite size (D) steadily drops from 59.31 nm to 50.38 nm. This decrease suggests that the addition of BaSrCa nanoparticles limits the crystalline development of HDPE, perhaps because of the increased number of nucleation sites and the obstruction of polymer chain mobility. The volume fractions show the intended composition trend: the HDPE fraction falls as the BaSrCa content rises from 4.89% to 19.25%. This verifies that the filler is evenly distributed throughout the polymer matrix in relation to its wt%.

As the concentration of ferrite increases, the lattice parameters of HDPE exhibit a tiny but steady reduction. In particular, lattice parameters a, b, and c fall from 7.418 Å to 7.410 Å, 4.940 Å to 4.933 Å, and 2.485 Å to 2.475 Å, respectively. These slight reductions in lattice dimensions imply that the interaction between the BaSrCa nanoparticles and HDPE chains causes modest stress or structural rearrangements in the polymer crystals. Improved packing efficiency or limited chain mobility brought on by the presence of stiff inorganic fillers are usually linked to such lattice contractions. Overall, the trends show that the BaSrCa nanoparticles affect the crystallization behavior of HDPE, resulting in a more compact, slightly constrained polymer structure that may improve the mechanical and thermal stability of the composites. These trends include decreasing crystallite size and lattice parameters with increasing filler loading.

**Fig. 2:**
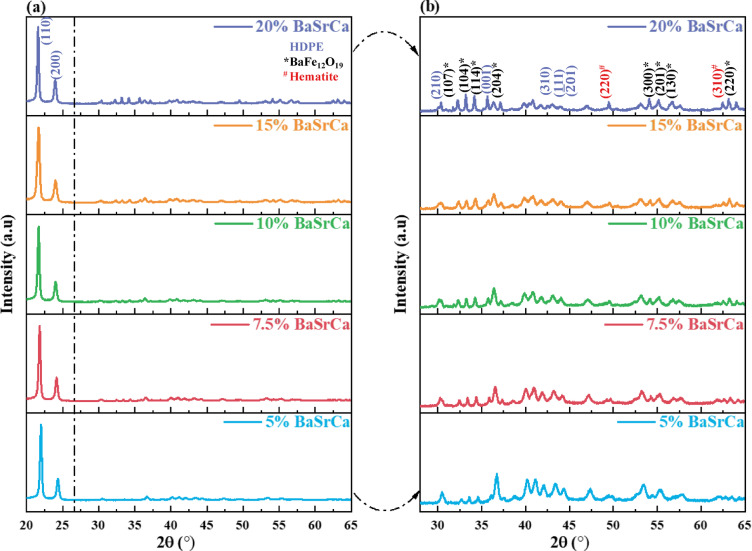
**a** XRD patterns of BaSrCa/HDPE composites and **b** zoom-in of the selected region.

The XRD analysis confirms the successful formation of BaSrCa/HDPE composites, as evidenced by the systematic evolution of diffraction patterns with increasing BaSrCa concentration (Fig. 2a). An examination of the 27°–65° angle range (Fig. 2b) reveals a clear reduction of HDPE peak intensities, simultaneous with a pronounced enhancement of BaSrCa peaks. This inverse intensity relationship signifies the effective dispersion and integration of the nanoparticles within the polymer matrix. The results show no variation of the HDPE characteristic diffraction peaks (2θ ≈ 21° and 24°) with the addition of nanoparticles. This indicated the preservation of the orthorhombic crystalline structure of the polymer after the addition of BaSrCa. The results are in good agreement with the reported literature for HDPE nanocomposites filled with various nanoparticles. HDPE systems reinforced with nano-graphite, graphene nanoplatelets, and carbon black, showed good crystallinity without new phases, and the nanoparticles acted as fillers and nucleating agents^15–17^. On the other hand, SiO_2_ and ZrO_2_ nanoparticles maintained the stability of the peak position of HDPE with an observed variation in peak intensity and sharpness^18^. This reflected the alteration in crystallinity and crystal size, mainly attributed to the enhanced interfacial interactions and nucleation at low addition of nanoparticle. At higher additions, the restriction of the chain mobility resulted with a slight decline in crystallinity. Hence, this confirms that the filling process via the nanoparticle incorporation impacts the structural features of HDPE without significantly altering its intrinsic crystallinity.

The distinctive HDPE peaks in the XRD patterns of the HDPE/BaSrCa composites progressively lose intensity as the BaSrCa loading increases, suggesting a decrease in HDPE crystallinity. On the other hand, when the filler percentage rises, the primary diffraction peaks of the BaSrCa hexaferrite phase become more noticeable, indicating the successful incorporation and growing contribution of the inorganic phase. Additionally, a minor shift and broadening of the HDPE peaks are visible, indicating weak lattice distortion and limited polymer chain mobility brought on by the interaction between HDPE and the BaSrCa nanoparticles. Thus, the evolution of the XRD patterns shows how the ferrite filler affects the polymer matrix structurally.

### TEM analysis

**Fig. 3:**
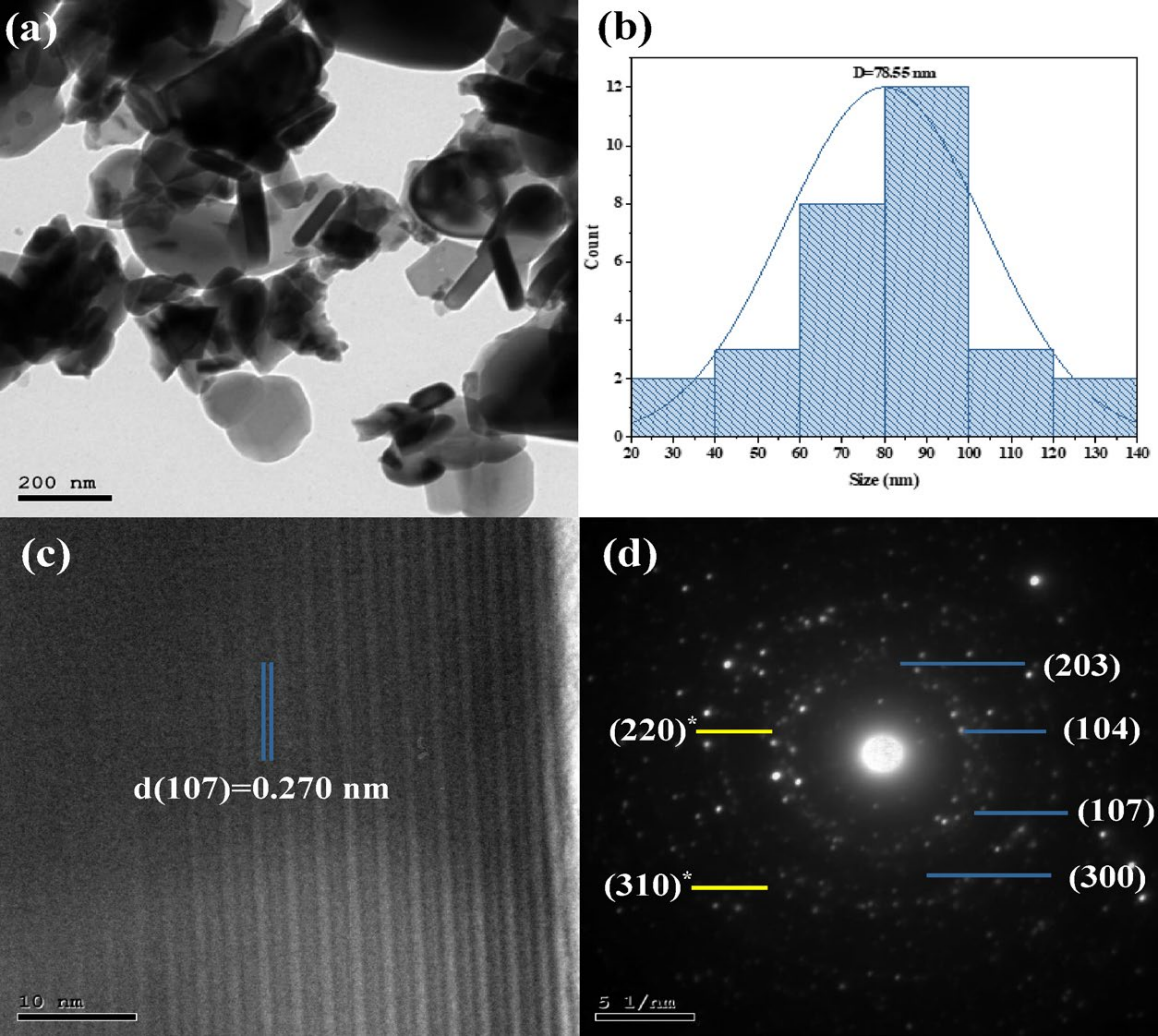
**a** TEM micrographs, **b** size distribution histogram, **c** HRTEM, and **d** SAED images for BaSrCa nanoparticles.

The morphological characteristics of the BaSrCa nanoparticles are illustrated in Fig. 3. TEM micrograph in Fig. 3a of the pure BaSrCa phase exhibits a well-defined hexagonal platelet-like morphology along with hexagonal pyramidal particles featuring oblique edges. This is consistent with previous reports in the literature^7^. The agglomeration of nanoparticles, evident in the TEM micrograph, arises from the strong magnetic interactions typical of ferrite-based nanostructured materials^8^. Statistical analysis of particle size distribution, performed using Image J software, indicated an average particle dimension of 78.55 nm. For more interpretation of the structural characteristics, high-resolution transmission electron microscopy (HRTEM) and selected area electron diffraction (SAED) investigations were conducted (Fig. 3a–d). HRTEM images display a distribution of grains with varying crystallographic orientations relative to the lattice planes, while the presence of well-defined lattice fringes confirms the high crystallinity of the nanoparticles. The polycrystalline structure is further corroborated by SAED patterns, which exhibit clear concentric rings corresponding to different (hkl) crystallographic planes. The interplanar spacing (d-spacing) was determined from SAED ring patterns using Image J software, with indexed reflections (203), (104), (107), and (300) corresponding to the BaSrCa phase, yielding a d-spacing of 0.270 nm. Moreover, the detection of (220) and (310) diffraction planes (Fig. 3d) confirmed the presence of hematite (α-Fe_2_O_3_) as a secondary phase in the sample. A comparative analysis of d-spacing values obtained from HRTEM and XRD data shows remarkable agreement, confirming the structural coherence of the synthesized nanoparticles.

### SEM-EDX Analysis

**Fig. 4:**
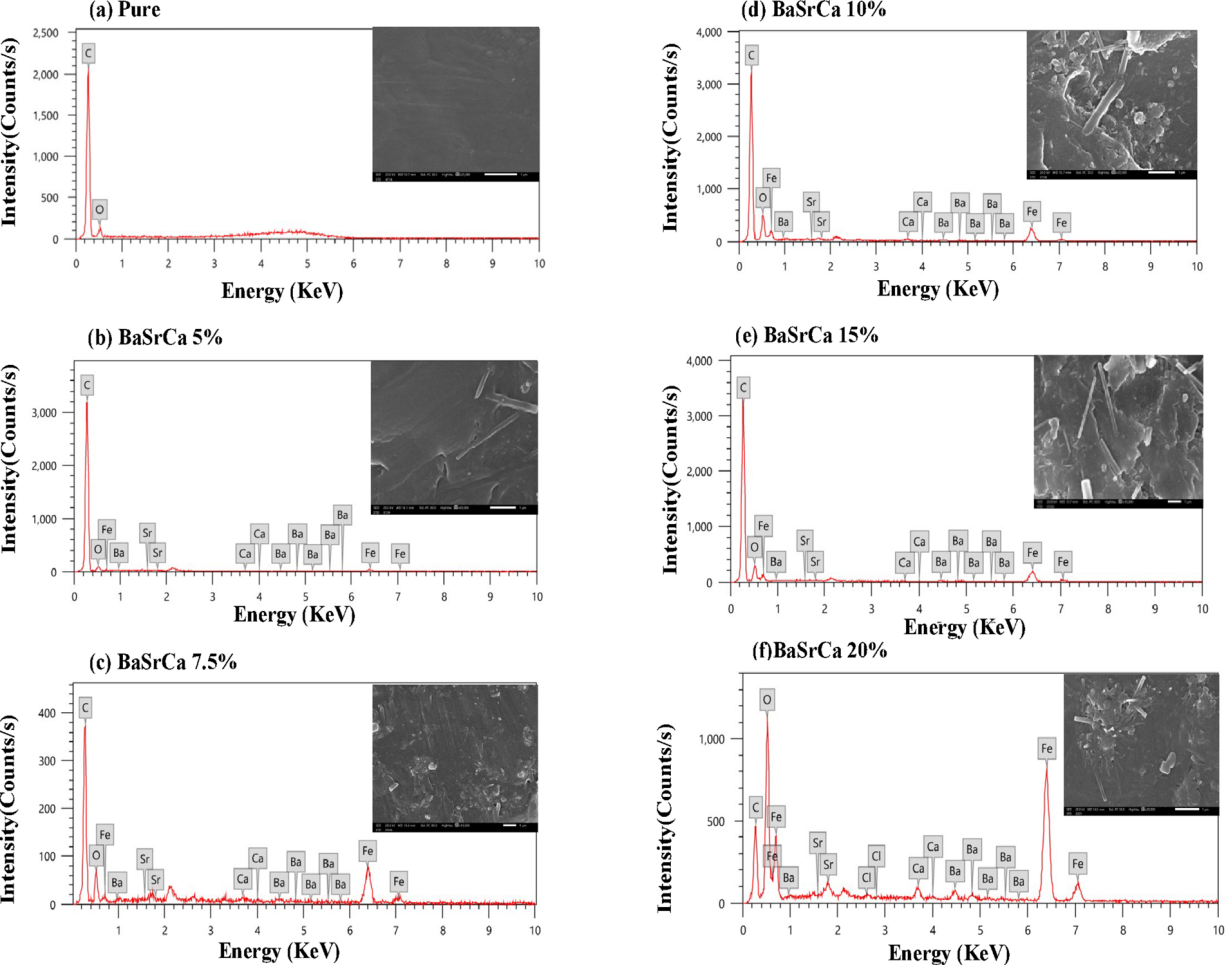
EDX of **a** Pure HDPE and **b**–**f** BaSrCa/HDPE composites with their SEM images as insets.

Scanning Electron Microscopy (SEM) micrographs were employed to investigate the microstructure of the synthesized samples. The obtained microstructural images reveal a plate-like morphology characterized by irregular agglomerations that coalesce to form larger particulate structures. These plate-like formations exhibit a homogeneous compositional contrast along with a smooth and featureless surface texture dispersed within a dominant matrix of larger, platelet-like hexagonal BaSrCa structures, as illustrated in Fig. 4.

The lack of inorganic elements and the purity of the polymer are confirmed by the EDX spectrum of the pure HDPE sample (Fig. 4a), which shows only the distinctive peaks of carbon and oxygen. The successful introduction of the multicomponent ceramic phase into the HDPE matrix is demonstrated by the appearance of new elemental peaks corresponding to Ba, Sr, Ca, and Fe in all composite samples (Fig. 4b–f) following the addition of BaSrCa fillers. The strength of the Ba, Sr, and Ca peaks is comparatively tiny but clearly discernible for low filler loadings (5–7.5%), indicating that the fillers are uniformly distributed throughout the polymer. The peaks of Ba and Ca become more noticeable as the filler percentage rises to 10–20%, indicating the increased concentration of these heavy elements. The presence of Fe peaks in several composites is attributable to either modest contamination from milling or processing equipment or to trace impurities in the ceramic powder. These peaks have little effect on the overall composition and stay low in comparison to the key elemental signals. The successful loading of BaSrCa fillers into the HDPE matrix is thus confirmed by the EDX results, which also corroborate the structural observations observed in the SEM micrographs. The durability of the filler and its uniform integration within the polymer framework are both confirmed by the consistent identification of distinctive elements across all composites and the increasing peak intensity at higher loadings.

### FTIR Analysis

**Fig. 5:**
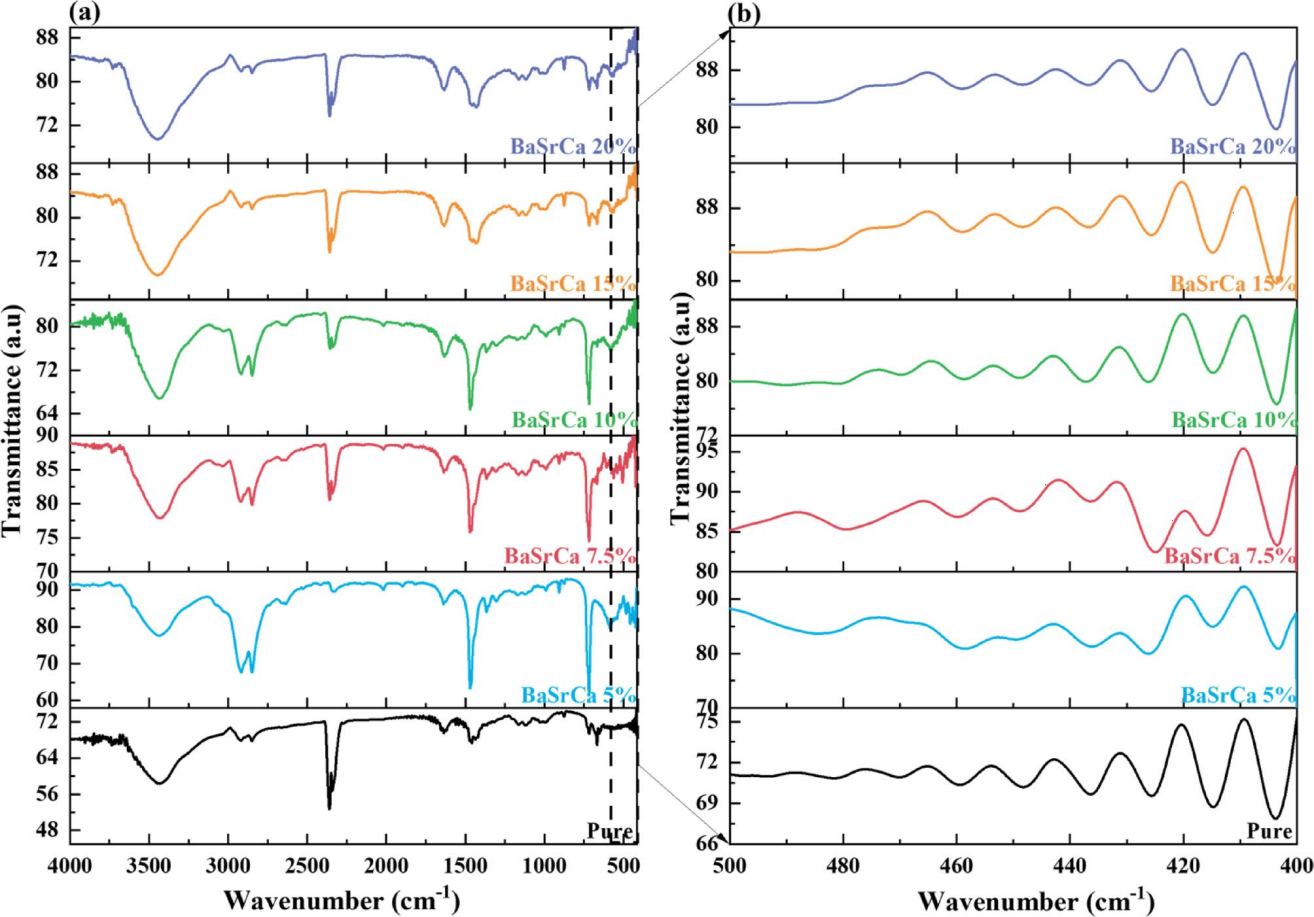
**a** Room temperature FTIR spectra of BaSrCa/HDPE composites and **b** zoom-in on the fingerprint region.

The Fourier transform infrared (FTIR) spectra of HDPE/BaSrCa composites, depicted in Fig. 5, were recorded across the wavenumber range of 400–4000 cm⁻¹. The analysis of the metal-oxygen region (Fig. 5b) reveals three distinct absorption bands positioned at 410–400 cm^−1^, 435–420 cm^−1^, and 595–579 cm^−1^, which are ascribed to the vibrational modes of tetrahedral and octahedral coordination groups. These bands confirm the existence of Fe–O and metal–oxygen (Ba–O, Sr–O) bonds within the tetrahedral and octahedral lattice sites, respectively^19^. In contrast, the FTIR spectrum of the neat HDPE displays the characteristic absorption bands at 2915 cm^−1^ (asymmetric –CH_2_– stretching) and 2819 cm^−1^ (symmetric –CH_2_– stretching). A bending vibration at 1470 cm^−1^ and a prominent peak at 722 cm^−1^ are associated with –CH_2_– deformation modes^19^. Notably, all principal peaks of HDPE remain unaltered in the nanocomposite spectrum, with no emergence of new absorption features or shifts in peak positions. This observation strongly indicates the absence of chemical interactions between the HDPE matrix and the ferrite fillers, thereby preserving the structural integrity of the composite system. For BaSrCa, two absorption bands are identified at υ_1_ = 582 cm^−1^ and υ_2_ = 436 cm^−1^. Certain bands demonstrate frequency variations, which may be attributed to modifications in the cation-oxygen (A–O) bond lengths within the nanoparticle structure. Additionally, a slight wavenumber shift toward higher frequencies is observed in some bands of the HDPE/BaSrCa composites, suggesting an enhanced crystallinity. This shift may arise from alterations in M–O bond lengths within the tetrahedral and octahedral sites. Also, it can be explained via the minor changes in lattice parameters (as deduced from XRD analysis). Consequently, bond length modifications directly impact the vibrational wavenumbers of the respective bonds.

Moreover, absorption peaks at 3443–3425 cm^−1^ (O–H stretching associated with adsorbed moisture) and 1641–1635 cm^−1^ (H–O–H bending) are present in all spectra, indicative of adsorbed water molecules^9^. Additionally, the peaks at 2924 cm^−1^ correspond to C–H stretching of CH_3_ groups, while a weak peak near 2200–2250 cm⁻^1^ is attributed to atmospheric CO_2_ contributions or weak overtone vibrations. Furthermore, C–O stretching vibrations are demonstrated by the observed peaks at 1040, 1050, and 1057 cm^−1^^20–22^.

### Tensile properties of BaSrCa/HDPE composites

**Fig. 6:**
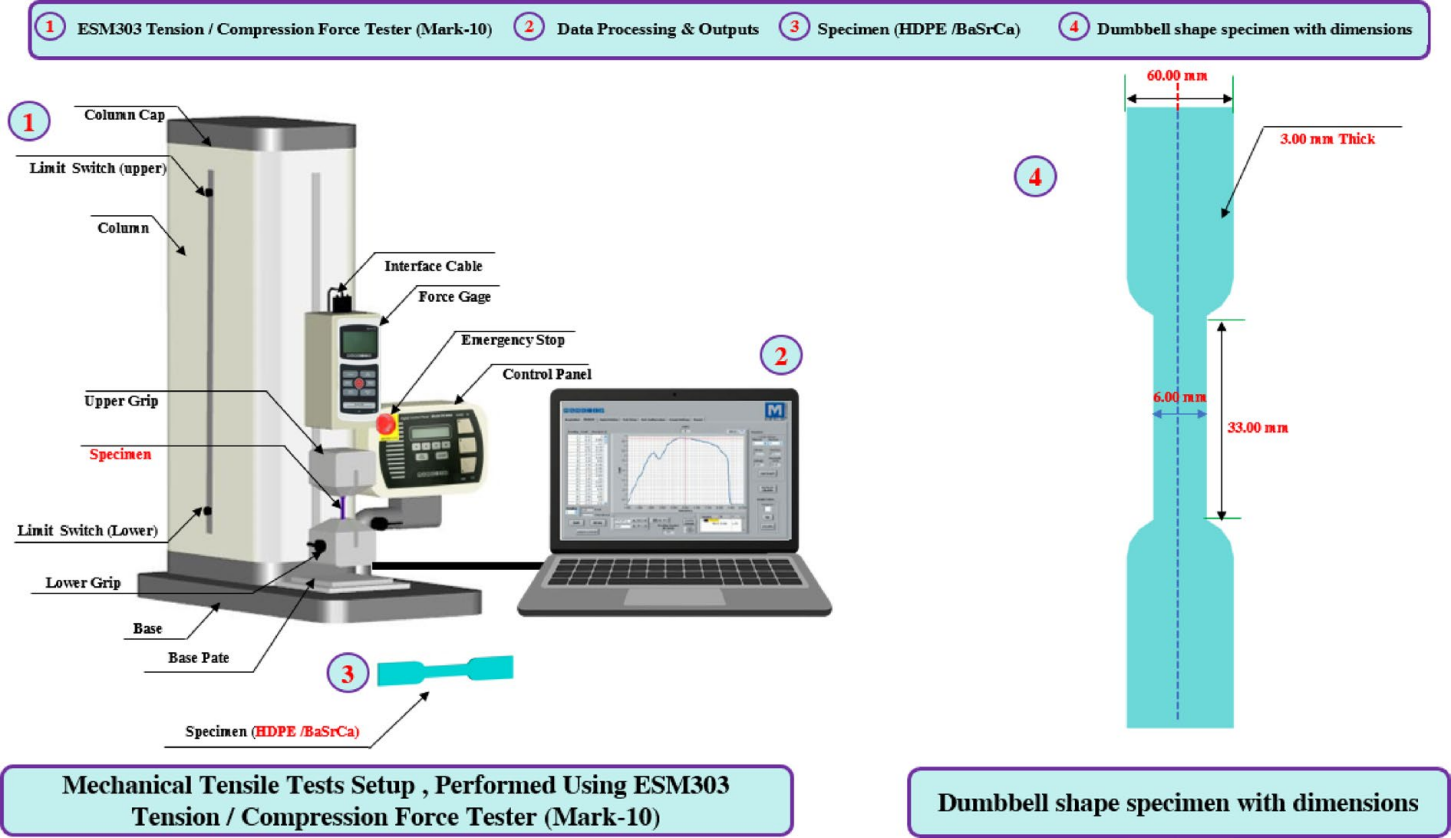
State-of-the-Art Tensile Testing Framework for HDPE/BaSrCa: Instrumentation and Specimen Geometry.

Using an ESM303 Tension/Compression Force Tester (Mark-10), the experimental setup for mechanical tensile testing of HDPE/BaSrCa composites is shown in Fig. 6. The schematic in the figure’s left panel highlights key components such as the control panel, force gauge, emergency stop, column cap, and upper and lower grips. Data acquisition and processing were carried out via a connected computer that recorded real-time stress–strain curves during testing to ensure precise and reliable measurements. The dumbbell-shaped specimen used in this study is shown in the figure’s right panel. It was carefully fabricated following the international standard ASTM D638, with dimensions of 3.00 mm thickness, 6.00 mm width at the narrow section, and 69.00 mm total length, ensuring uniformity and consistency across all tests. This standardized geometry provided accurate measurements of tensile properties, including tensile strength and elongation at break.

Tensile tests were conducted at room temperature using crosshead speeds of 5 mm/min, 10 mm/min, and 20 mm/min for each weight fraction of BaSrCa filler.

**Fig. 7:**
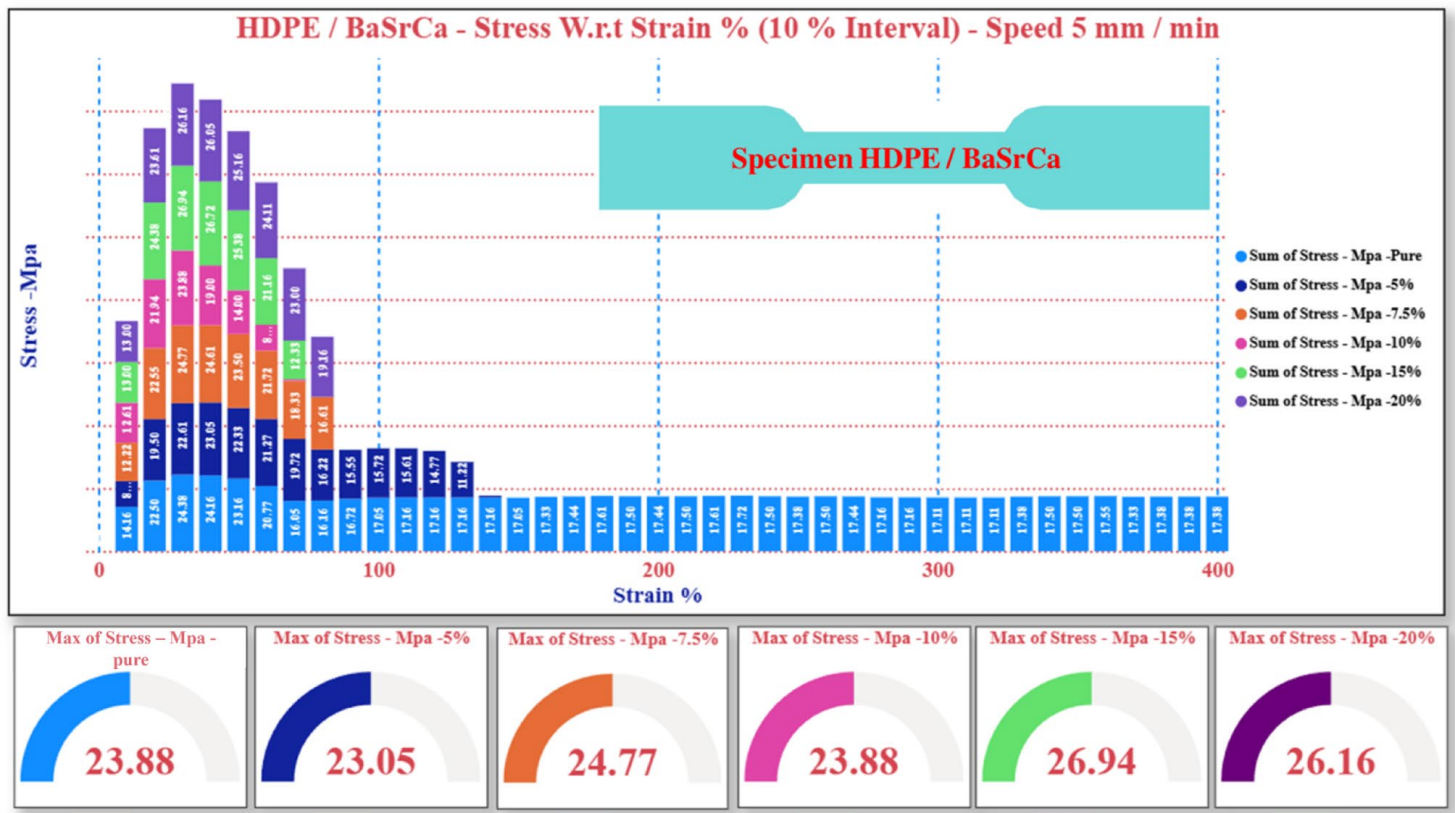
The stress-strain behavior of BaSrCa/HDPE composites reinforced with (0, 5, 7.5, 10, 15, and 20 wt%) at 5 mm/min.

**Fig. 8:**
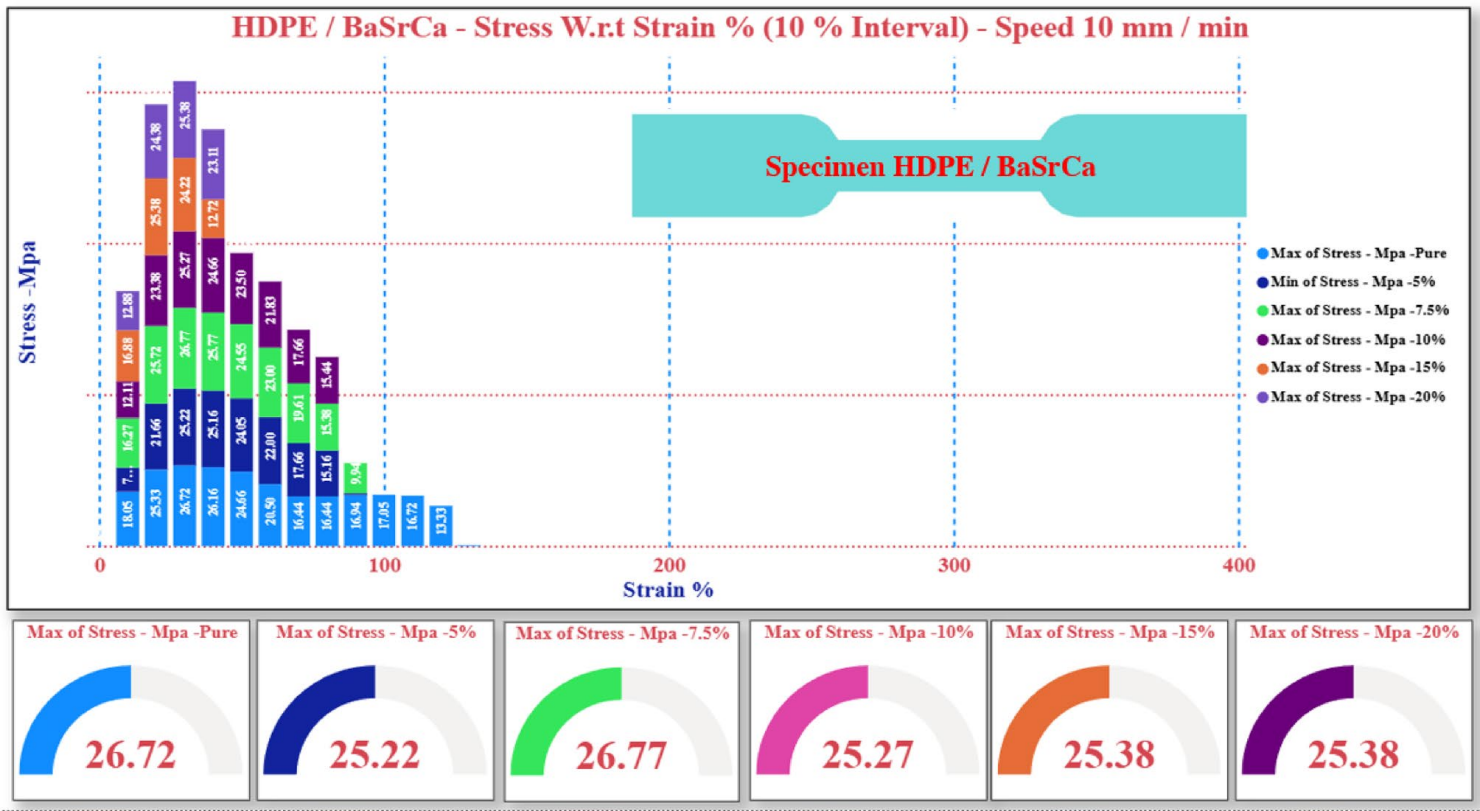
The stress-strain behavior of BaSrCa/HDPE composites reinforced with (0, 5, 7.5, 10, 15 and 20 wt%) at 10 mm/min.

**Fig. 9:**
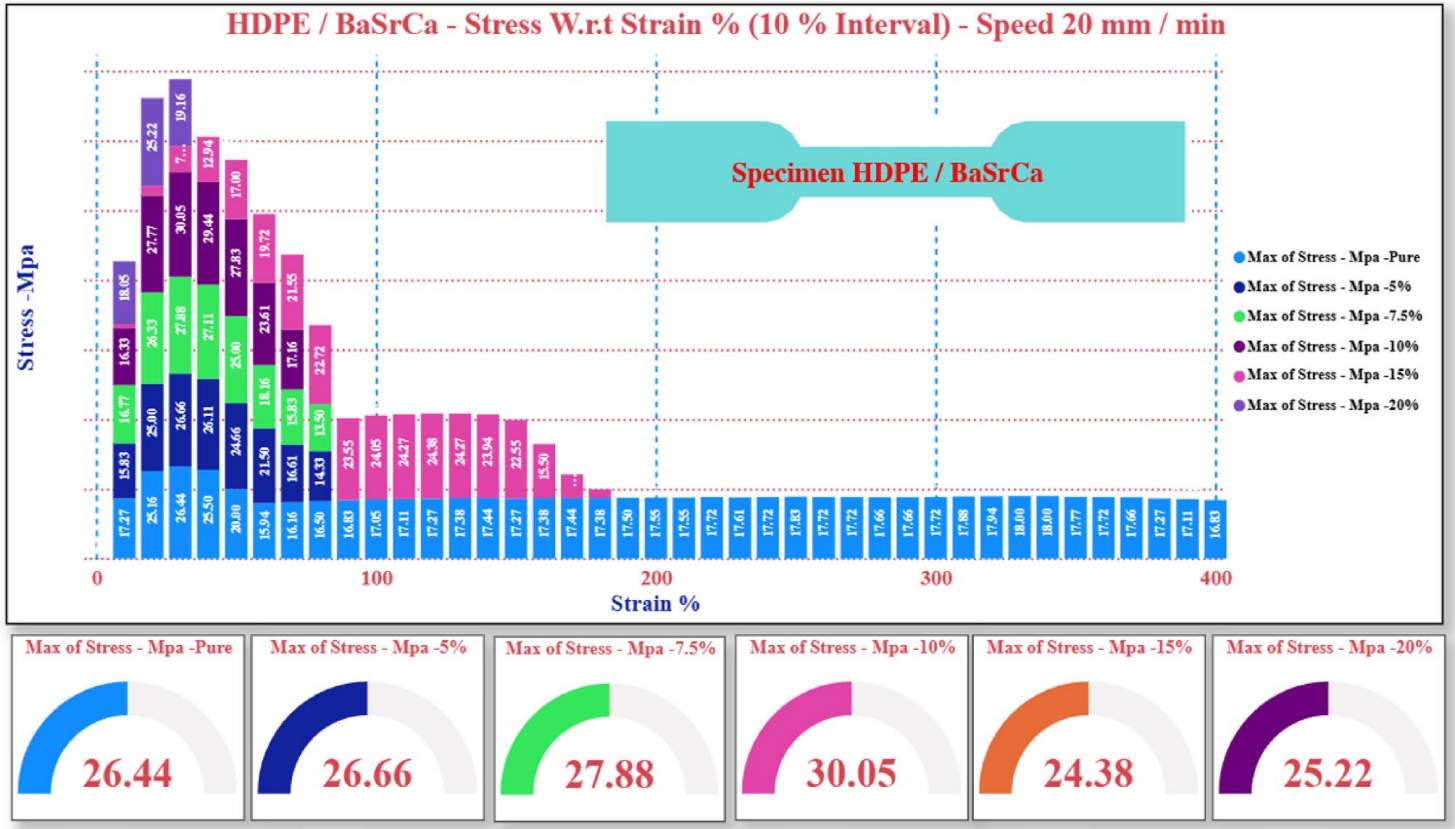
The stress-strain behavior of BaSrCa/HDPE composites reinforced with (0, 5, 7.5, 10, 15, and 20 wt%) at 20 mm/min.

The stress-strain behavior of HDPE composites was displayed as a function of BaSrCa wt%, at each strain rate, in Figs. 7, 8, and 9, respectively. Tables 3, 4, and 5 list the mechanical parameters of the different strain rates 5, 10, and 20 mm/min, respectively, including the tensile stress in MPa and the resulting strain in %, the toughness, and the modulus of resilience. The incorporation of BaSrCa nanoferrite fillers into the HDPE matrix led to substantial modifications in the tensile behavior across all strain rates. Young’s modulus increased consistently with rising filler content, demonstrating improved stiffness due to the rigid nature of BaSrCa particles and their effective interaction with the HDPE matrix. At 5 mm/min, Young’s modulus values increase from 144.35 MPa for pure HDPE to 191.35 MPa at 20 wt%. Similar upward trends were recorded at 10 mm/min and 20 mm/min, reaching 218.59 MPa and 239.04 MPa, respectively. These improvements are consistent with the effect of stiff ceramic particles in enhancing the elastic modulus and yield stress by restricting chain mobility and better load transfer^23,24^. The yield stress behavior revealed a complex and informative pattern with respect to both filler content and strain rate. At the lowest strain rate, 5 mm/min, yield stress increased from 16.48 MPa for pure HDPE to 25.37 MPa at 20 wt% BaSrCa. At intermediate and higher strain rates, yield stress continued to increase more smoothly, peaking at 28.62 MPa for 10 wt% BaSrCa at 20 mm/min. This trend highlights a viscoelastic–plastic transition, where increased strain rate suppresses relaxation mechanisms and allows stiffer filler networks to dominate the mechanical response^25,26^. Interestingly, unlike the 5 mm/min case, the 20 mm/min regime showed less performance degradation at high filler content. This explains that rapid loading limits the development of localized strain softening and crack initiation at particle agglomerates^27,28^.

**Table 3:** The mechanical properties of BaSrCa/HDPE composites reinforced with (0, 5, 7.5, 10, 15, and 20 wt%) at 5 mm/min.

Sample	Speed: 5 mm/min							
	E (MPa)	UTS (MPa)	Yield strain (%)	Yield stress (MPa)	Strain at break (%)	Stress at break (MPa)	Toughness (MJ/m^3^)	Modulus of resilience (MJ/m^3^)
Pure	144.35	24.44	11.6	16.48	536.01	16.72	2073.63	0.97
BaSrCa 5%	148.21	24.51	15.76	17.05	150.24	0.55	167.27	1.84
BaSrCa 7.5%	166.24	24.88	15.84	20.78	90.84	0.53	68.60	2.08
BaSrCa 10%	169.36	24.98	15.96	21.48	75.63	0.5	48.44	2.15
BaSrCa 15%	178.94	27.05	17.61	24.01	73.5	0.44	48.33	2.77
BaSrCa 20%	191.35	27.33	20.5	25.37	69.87	0.33	46.71	4.02

**Table 4:** The mechanical properties of HDPE/BaSrCa composites reinforced with (0, 5, 7.5, 10, 15 and 20 wt%) at 10 mm/min.

Sample	Speed: 10 mm/min							
	E (MPa)	UTS (MPa)	Yield strain (%)	Yield stress (MPa)	Strain at break (%)	Stress at break (MPa)	Toughness (MJ/m^3^)	Modulus of resilience (MJ/m^3^)
Pure	149.78	26.72	9.64	18.17	129.76	0.53	126.09	0.69
BaSrCa 5%	173.77	26.78	14.24	18.49	117.52	0.5	119.99	1.76
BaSrCa 7.5%	176.88	26.92	15.76	23.44	109.73	0.45	106.48	2.19
BaSrCa 10%	195.39	27.27	19.58	23.45	102.55	0.44	102.74	3.74
BaSrCa 15%	206.46	27.31	19.76	24.38	52.99	0.41	28.98	4.03
BaSrCa 20%	218.59	28.05	19.86	24.68	50.69	0.38	28.08	4.31

**Table 5:** The mechanical properties of HDPE/BaSrCa composites reinforced with (0, 5, 7.5, 10, 15, and 20 wt%) at 20 mm/min.

Sample	Speed: 20 mm/min							
	E (MPa)	UTS (MPa)	Yield strain (%)	Yield stress (MPa)	Strain at break (%)	Stress at break (MPa)	Toughness (MJ/m^3^)	Modulus of resilience (MJ/m^3^)
Pure	178.32	26.74	16.69	24.32	571.76	14.17	2914.72	2.48
BaSrCa 5%	194.03	26.86	17.62	24.42	90.54	0.5	79.52	3.01
BaSrCa 7.5%	207.59	27.88	18.65	25.83	85.87	0.44	76.53	3.61
BaSrCa 10%	226.84	28.05	19.58	28.62	77.15	0.38	67.50	4.34
BaSrCa 15%	227.73	28.38	21.43	22.77	48.97	0.35	27.30	5.22
BaSrCa 20%	239.04	28.61	13.91	24.29	31.27	0.31	11.68	2.31

The ultimate tensile strength (UTS) of HDPE composites reinforced with BaSrCa nanoferrites demonstrates modest yet consistent enhancement with increasing filler content across all strain rates. At 5 mm/min, the tensile strength increases from 24.44 MPa for pure HDPE to 27.33 MPa at 20 wt% BaSrCa. The same behaviour was observed at 10 mm/min, where UTS improved from 26.72 MPa for pure HDPE to 28.05 MPa for 20 wt% BaSrCa, and at 20 mm/min from 26.74 MPa for pure HDPE to 28.61 MPa for the 20 wt% BaSrCa. These improvements indicate that stiff nanoferrite fillers increase the load transfer efficiency, which improves the maximum stress to failure capacity of the composite^29^. However, the magnitude of improvement is relatively modest, which may be attributed to a balance between reinforcement and embrittlement effects as filler loading increases^30^.

The strain at break, conversely, exhibits a pronounced reduction with increasing BaSrCa content. At a strain rate of 5 mm/min, the strain at break decreased from 536.01% for pure HDPE to 69.87% at 20 wt% BaSrCa. This downward tendency was further followed at 10 mm/min from 129.76% of pure HDPE to 50.69% at 20 wt% BaSrCa, and at 20 mm/min from 571.76% for pure HDPE down to 31.27% at 20 wt% BaSrCa. However, this significant diminution suggests a decrease in the ductility, which is frequently observed in polymer nanocomposites with high filler loading. The mobility of the polymer chains becomes limited, and the premature failure at the filler–matrix interface is generated^31^. The contrast between UTS enhancement and strain at break reduction reflects the dual role of BaSrCa fillers, which reinforce the matrix and improve peak stress capacity yet compromise the material’s ability to undergo plastic deformation. Such trade-offs are typical of highly crystalline polymer-filler systems where filler-induced stiffening dominates at the expense of ductility^28,32^. At 20 mm/min strain rates, the decline in strain at break becomes less dramatic, especially beyond 10 wt%, suggesting that rapid loading mitigates crack initiation at filler clusters. This observation reinforces the viscoelastic nature of HDPE and the rate-dependent failure modes reported in similar studies^26,33^.

Toughness and modulus of resilience revealed non-linear behaviors that depend on the interplay between elastic storage and plastic dissipation. At low strain rates, toughness decreased sharply with filler loading, reflecting reduced elongation at break. In contrast, modulus of resilience, which indicates a measure of elastic energy storage, showed a significant increase with higher BaSrCa content, especially at higher strain rates like 5.22 MJ/m^3^ at 15 wt% and 20 mm/min. This reflects improved energy storage in the elastic regime, despite diminished plastic deformation capacity^34^. These findings suggest that the BaSrCa nanoferrite particles are particularly effective in enhancing the elastic region properties of HDPE composites under dynamic loading, with implications for applications requiring high stiffness and moderate toughness.

Based on the mechanical performance trends across the tested strain rates, the optimal weight fraction of BaSrCa nanoferrites in HDPE composites appears to be in the range of 10–15 wt%, depending on the target property. At 10 wt%, the composite demonstrates a well-balanced enhancement in Young’s modulus, yield stress, and UTS across all strain speeds. For instance, at 20 mm/min, Young’s modulus increased to 226.84 MPa from 178.32 MPa for pure HDPE, and yield stress peaked at 28.62 MPa, the highest value observed in the dataset. This suggests effective load transfer due to good filler dispersion in the polymer matrix, without significant agglomeration that could compromise mechanical integrity. At 15 wt%, further gains were observed in modulus of resilience up to 5.22 MJ/m^3^ at 20 mm/min, indicating improved energy absorption in the elastic range. However, accompanying reductions in strain at break and toughness, especially at lower strain rates, indicate the onset of embrittlement behavior likely due to filler agglomeration and reduced polymer chain mobility. These observations are consistent with findings from similar nanofiller-reinforced HDPE systems. Zebarjad et al.^33^ reported diminishing returns and ductility losses beyond 10 vol% CaCO₃, citing filler agglomeration as a critical limiting factor. Alsayed et al.^12^ also noted that ZnFe₂O₄ fillers significantly enhanced modulus and yield stress up to 15 wt%, after which mechanical uniformity was compromised. The 20 wt% composites, while exhibiting the highest stiffness, showed marked reductions in ductility and toughness across all strain rates, especially pronounced at 5 mm/min, where strain at break dropped to just 69.87%. This suggests that beyond 15 wt%, particle agglomeration dominates, acting as failure initiation sites under tensile loading^32,35^. These results affirm the general understanding that optimal nanoparticle loading in polymer composites typically lies within 5 to 15 wt%, depending on particle dispersion, and testing strain rate^26,28^.

### Thermal properties using thermal gravimetric analysis (TGA)

**Fig. 10:**
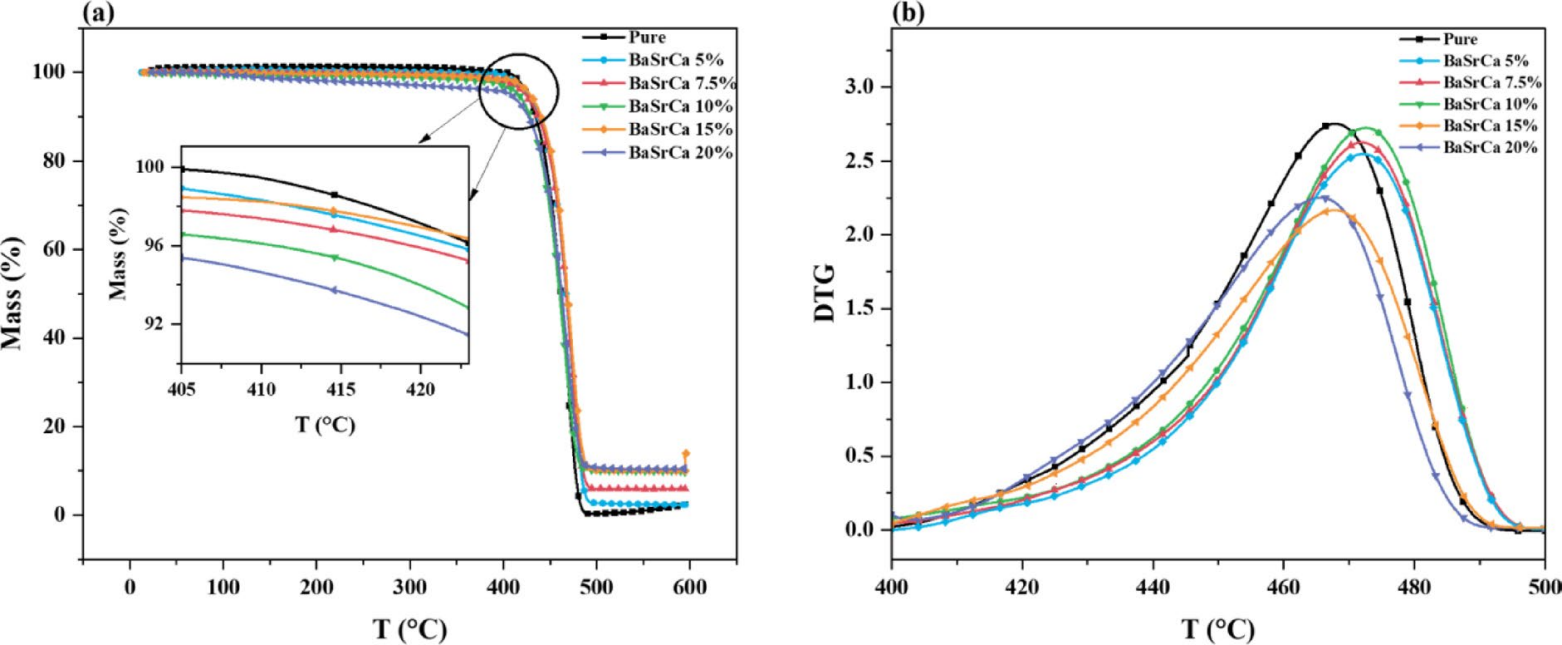
**a** TGA curves for HDPE/BaSrCa nanoparticles and **b** DTG curves for the same group of samples.

Thermogravimetric and derivative thermogravimetric analyses were used to assess HDPE’s heat resilience. A comparison of the BaSrCa system’s impact on HDPE’s thermal properties is shown in Fig. 10a. The pertinent derivatives (DTG) are shown in Fig. 10b. Table 6 displays the residual mass percentage as well as the T_5%_ and T_50%_ temperatures, which represent a 5% and 50% weight loss of the samples under examination, respectively. There was just one deterioration phase visible in the TGA curves for every sample. With disintegration starting at 425.85 °C and continuing until total breakdown at 461.67 °C, pure HDPE displayed a 2.41% residual mass. According to comparable findings by Contat et al.^36^, the TGA data indicated that HDPE’s heat deterioration began at roughly 390 °C, with the maximum degradation rate near 474 °C and complete decomposition by ~ 502 °C.

When 5% to 20% of BaSrCa hexaferrite nanoparticle reinforcements are added, the thermal stability of HDPE is significantly improved compared to the pure material. As the concentration of these nanofillers increases to 20%, the initial degradation temperature (T_5%_) drops from 425.85 °C to 407.79 °C. Additionally, T_50%_ values for BaSrCa/HDPE composites exhibit the same trend as T_5%_, decreasing from 461.67 °C to 457.52 °C as the BaSrCa nanofiller concentration increases to 20%.

**Table 6:** Thermal degradation properties of HDPE polymer composites with BaSrCa nanoparticles.

Sample	TGA results			DTG results	
	T_5%_ (°C)	T_50%_ (°C)	Residual mass %	Decomp. T (°C)	Decomp. Rate (%/°C)
Pure	425.85	461.67	2.41	468.19	2.75
BaSrCa 5%	422.29	460.99	2.96	471.93	2.54
BaSrCa 7.5%	421.90	459.55	5.95	472.12	2.63
BaSrCa 10%	416.42	459.15	9.94	472.71	2.72
BaSrCa 15%	415.55	458.68	9.97	467.92	2.16
BaSrCa 20%	407.79	457.52	10.51	465.09	2.25

Through several synergistic processes, embedding BaSrCa hexaferrite nanoparticles into HDPE can lower both T_5%_ and T_50%_ degradation temperatures. By proactively breaking polymers into smaller pieces, the iron-based oxides included in the nanoparticles are known to function as catalysts for heat degradation. Additionally, the nanoparticles partially block HDPE’s crystalline structure, allowing for a higher percentage of amorphous regions to exist and break down at lower temperatures. Furthermore, it would also be feasible to introduce interfacial flaws, which are thermally weak spots. As a result of the expedited onset and quicker breakdown progression in nanocomposite systems, T_5%_ and T_50%_ values both decrease. Similar results were reported by Munoz et al.^37^, who showed that the catalytic degrading activity of iron oxides in polyolefin matrices was demonstrated by reducing T_5%_ from 433 °C to 384 °C and T_50%_ from 478 °C to 409 °C by adding γ-Fe_2_O_3_ nanoparticles into HDPE^37^. Furthermore, Alateyah^38^ claimed that adding 2.5 wt% graphite and 2 wt% magnesium oxide to the HDPE matrix resulted in a little drop in the onset temperature from 465 °C to 464 °C. This was ascribed to a weaker adherence of the filler-matrix interface^38^.

Additionally, Table 6 shows that residual mass increases after the deterioration phase concludes. As the nanofiller content rises to 20%, it increases from 2.41% to 10.51%. This implies that while avoiding total collapse, the composite structure promotes the development of char. The nano-sized fillers appear to provide even greater stability due to their increased surface area and stronger contact with the polymer matrix. Well-dispersed nanoparticles enhance polymer-filler interfacial adhesion and provide better heat shielding, both of which improve thermal resistance, according to Majka et al.^39^.

The thermal stability of the materials is indicated by the DTG data, which shows a variety of decomposition temperatures, mainly clustered around 465–473 °C. Although there are variances, these comparatively near results imply that the main decomposition event for these materials takes place within a particular temperature range. The peak decomposition temperature appears to rise slightly from 468.19 °C to 472.71 °C at small concentrations (5%, 7.5%, and 10%), indicating a little improvement in stability or a change in the decomposition mechanism. This is in line with the widespread pattern of increased thermal resistance following filler addition. The peak temperature appears to drop to 465.09 °C at greater concentrations (15% and 20%), suggesting a possible instability or an alternative breakdown route.

The range of maximal decomposition rates, which range from 2.16%/°C to 2.75%/°C, indicates that the composition influences the degradation kinetics. At greater concentrations of 15% and 20% BaSrCa, however, the maximum rate of breakdown decreases. This might be because the ingredient is a diluent, char promoter, or changes the chemical pathways of degradation, causing a slower or more gradual mass loss. These results align with Osman et al.^40^, who claimed that upon the addition of lead oxide nanoparticles with 35 wt%, the decomposition rates decreased from 3.06%/°C to 2.26%/°C, thus indicating an increase in the thermal stability of polystyrene polymer.

A kinetic analysis of the thermal degradation process for pure HDPE and HDPE/BaSrCa composites has been carried out to completely comprehend the degrading behavior of composites in contrast to pure HDPE. The activation energy, $\:E,\:$can be calculated using Eq. (2) and the approximations given by the subsequent equation^41^.

2 $$\:ln\left({ln}\left(\frac{m}{{m}_{0}}\right)\right)=-\frac{E}{R}\left(\frac{1}{T}\right)+A,$$

where $\:A$ is the frequency of molecular collisions that result in decomposition, $\:R$ is the ideal gas constant, or 8.314 J/mol⋅K, and $\:m$ and $\:{m}_{0}$ are the masses of the sample at a particular temperature and the initial sample mass, or 100%, respectively.

**Fig. 11:**
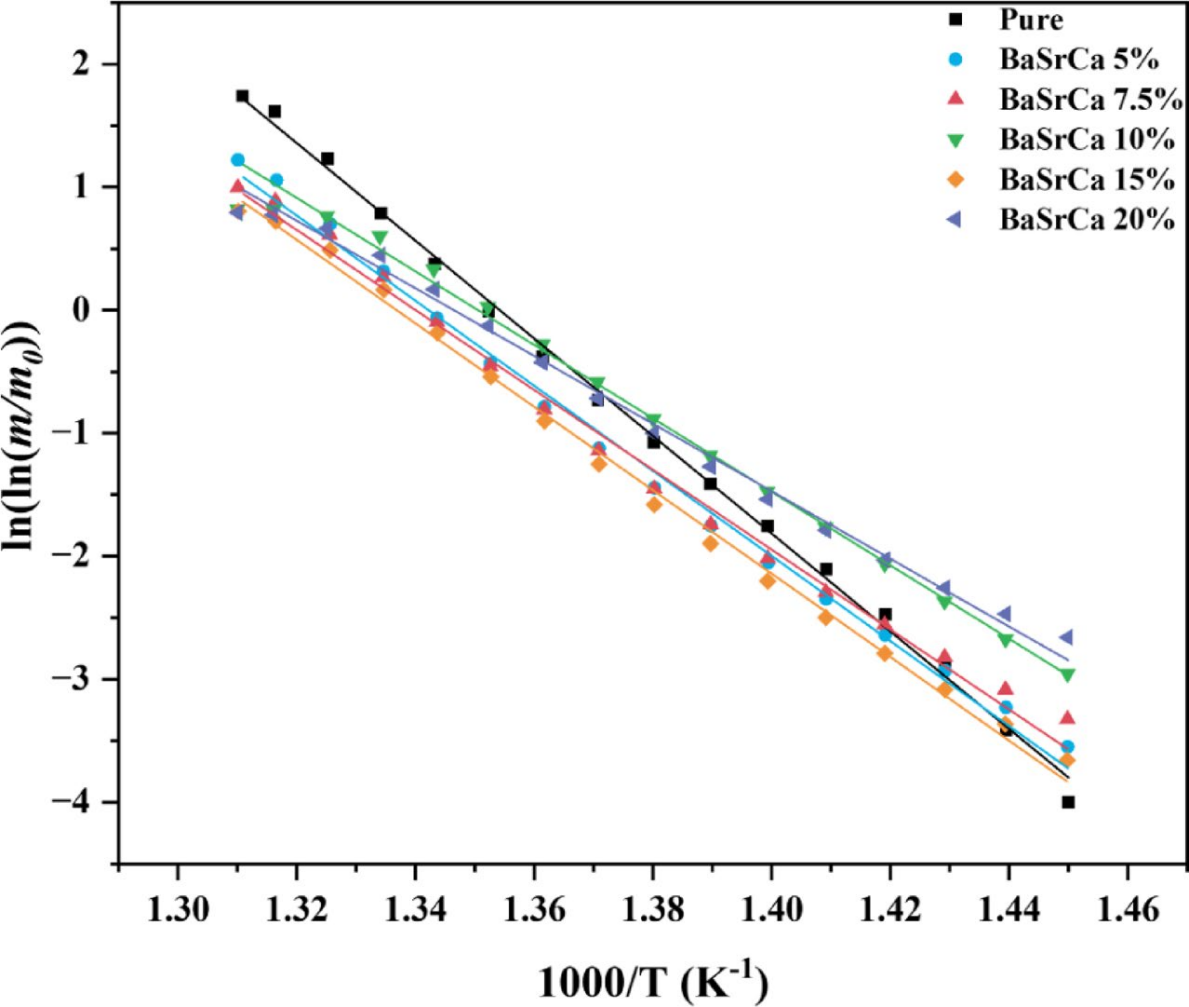
$\:ln({ln}\left(\frac{m}{{m}_{0}}\right))$ against $\:\frac{1}{T}$ of HDPE/BaSrCa nanoparticles.

**Fig. 12:**
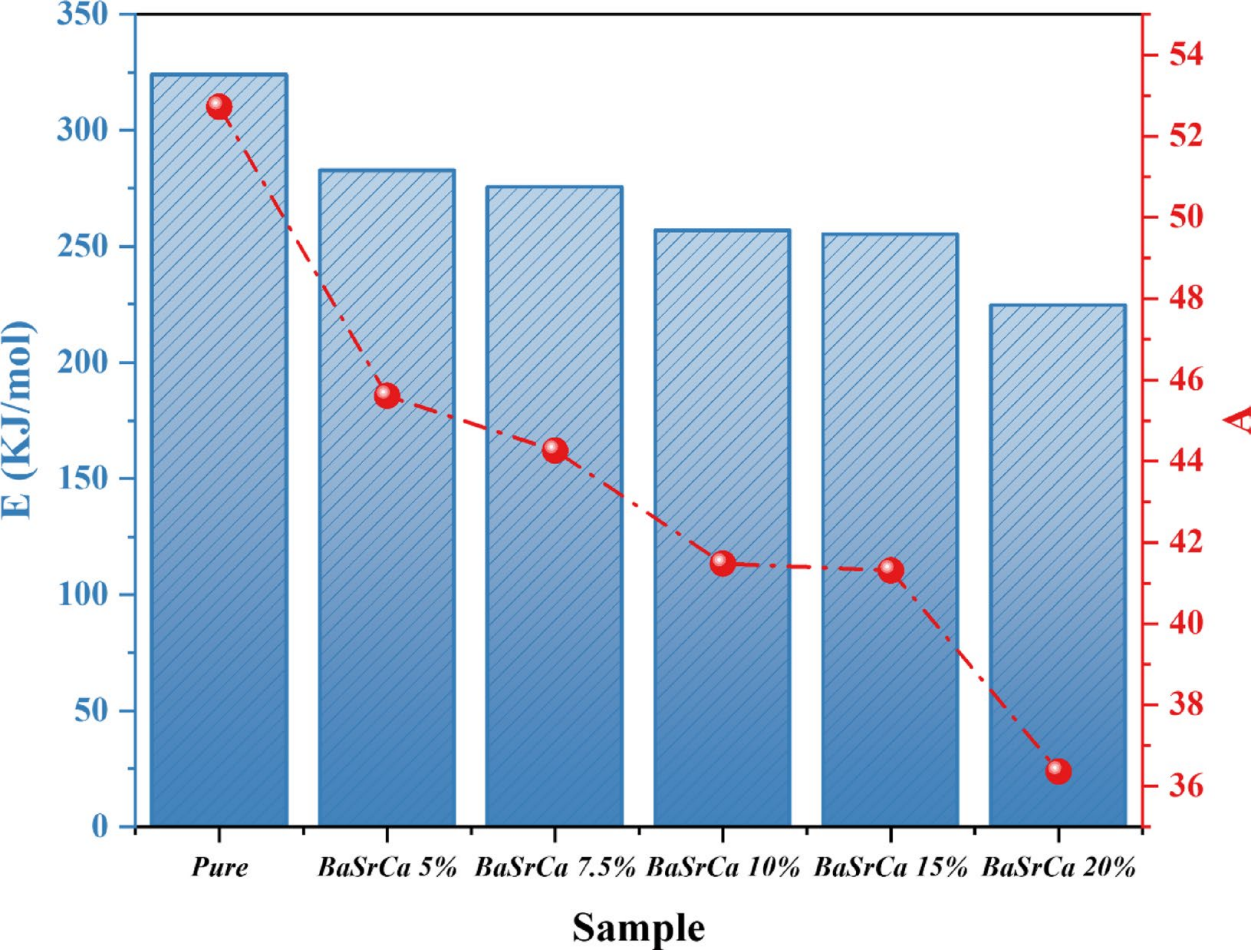
Kinetic parameters calculated for HDPE polymer composites with BaSrCa nanoparticles.

For this, the TGA data of the HDPE/BaSrCa under examination were utilized. The $\:ln({ln}\left(\frac{m}{{m}_{0}}\right))$ plots against 1/T in Fig. 11, thus yielding a straight line with a slope of $\:-\frac{E}{R}$. Thus, the activation energy $\:E$ was determined using the slope. Figure 12 displays the kinetic properties of all the prepared samples. The lines at different HDPE/BaSrCa composite weight fractions are found to be approximately parallel to one another. Furthermore, each activation energy’s linearity correlation coefficient is higher than 0.99, indicating that the degradation process is sufficiently described by the kinetic model in use.

As the concentration of nanofiller in HDPE/BaSrCa increases, Fig. 12 clearly illustrates a declining trend in $\:E$ and $\:A$. According to tests on activation energy, these nanofillers reduce the degradation energy barrier. Filler-induced heterogeneities that serve as heat transfer sites and hasten deterioration may be the cause of the lower activation energy. Weak connections in polymer composites, like head-to-head, hydroperoxy, and peroxy structures, are connected to polymer degradation because they easily degrade at very low temperatures to produce radicals that speed up the degradation process at higher temperatures. The residual mass measurements from TGA show better char development despite the slope’s decline, which enhances thermal protection^40^. This bolsters the idea that nanofillers improve thermal barriers and postpone breakdown by lowering heat transmission and polymer chain mobility. Particularly at increasing nanofiller concentrations, the $\:A$ factor exhibits a declining trend, suggesting a decreased probability of spontaneous degradation events (Fig. 12). The idea is that nanosized fillers improve the thermal barrier effect by preventing heat-induced polymer breakdown and encouraging a more homogeneous dispersion. Comparable patterns in the literature suggest that nano-fillers, like lead oxide nanoparticles, enhance the interfacial interaction between the polymer and the filler, increasing the thermal stability of the polystyrene polymer^40^. These nanoparticles improve overall thermal stability by delaying the deterioration process through enhanced interfacial adhesion and heat dissipation effects.

### Thermal properties using Differential Scanning Calorimetry (DSC)

**Fig. 13:**
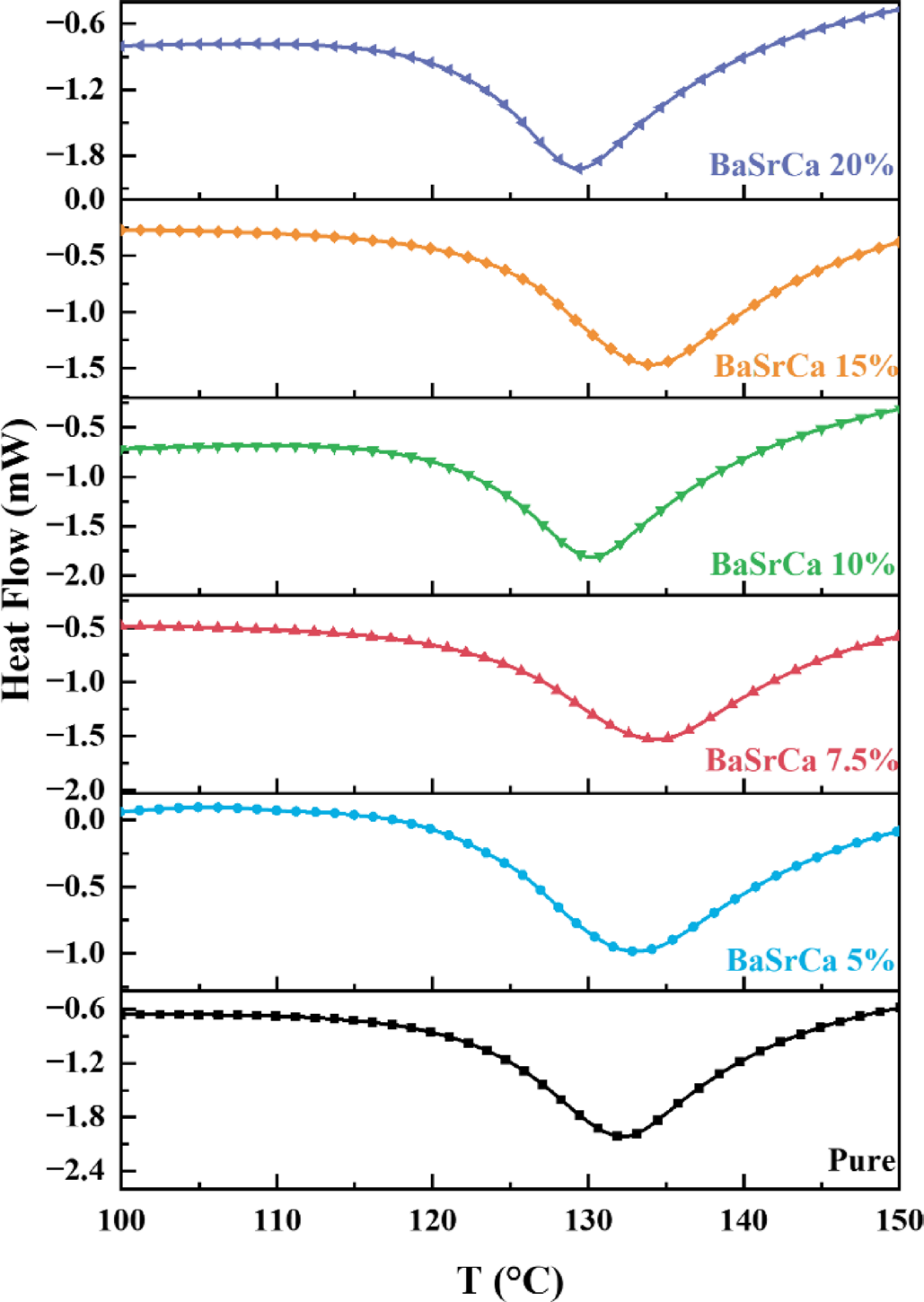
DSC heating curves for HDPE/BaSrCa nanoparticles.

The effects of BaSrCa nanofillers on the thermal behavior of HDPE are thoroughly understood from the Differential Scanning Calorimetry (DSC) thermograms and associated thermal transition data. Figure 13 shows the DSC curves of HDPE/BaSrCa composites.

One common semicrystalline polymer is HDPE. To the best of our knowledge, both semicrystalline and amorphous polymers undergo a glass transition when the temperature rises. But semicrystalline polymers, like HDPE, crystallize and melt, whereas amorphous polymers just show the glass transition. HDPE has a glass transition temperature; however, it is not very high, and thermal studies frequently fail to identify it. The glass transition temperature of HDPE is between − 130 °C and − 100 °C; above this range, HDPE becomes brittle because of reduced molecular mobility, according to Greene et al.^42^.

**Fig. 14:**
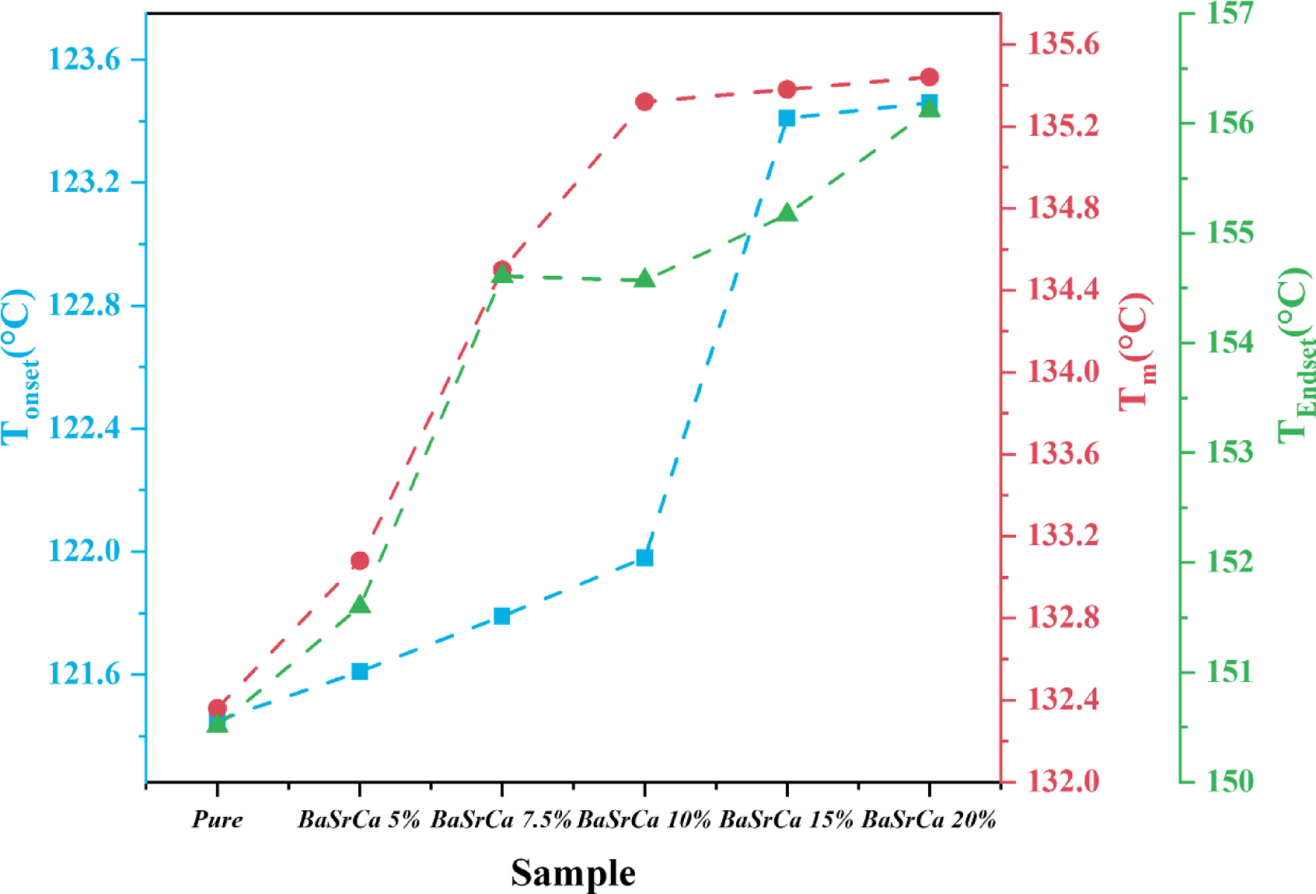
DSC results calculated for HDPE polymer composites with BaSrCa nanoparticles.

The thermal curves in this study primarily display transitions associated with melting and crystallization, with no observable glass transition temperature within the 100–170 °C temperature range under investigation. The behavior of HDPE aligns with this, as its glass transition temperature of approximately − 100 °C is significantly lower than the scanning range utilized. The melting temperatures at onset ($\:{T}_{onset}$), peak ($\:{T}_{m}$), and endset ($\:{T}_{Endset}$) are among the DSC measurement results presented in Fig. 14.

The $\:{T}_{onset}$ and $\:{T}_{Endset}$ of the HDPE pure sample are 121.45 °C and 150.51 °C, respectively, whereas its $\:{T}_{m}$ is 132.36 °C. The increase is evident with the addition of BaSrCa nano fillers, as shown in Fig. 14. When these nanofillers are used, the $\:{T}_{onset}$, which increases to 123.46 °C for the BaSrCa 20% sample. Additionally, $\:{T}_{m}$ and $\:{T}_{Endset}$ exhibit the same pattern, rising to 135.44 °C and 156.12 °C, respectively. Numerous factors contribute to the observed increase in these values. First and foremost, these nanoparticles act as efficient heterogeneous nucleating agents, promoting the alignment of polymer chains and facilitating the formation of more ordered crystalline regions, both of which raise the composite’s melting point and thermal stability. Research has shown that by providing nucleation sites, nanoparticles enhance chain ordering and increase melting transitions in semicrystalline polymers^43^. Additionally, due to their ceramic-like properties, BaSrCa nanoparticles contribute to a thermal barrier effect that impedes heat transport through the polymer matrix and reduces chain mobility; this thermal resistance delays the melting process^44^. This lends credence to the notion that nanofillers’ high surface area and robust interactions with the polymer matrix make them more potent nucleating agents. Their ability to refine the crystalline structure leads to higher thermal stability in the melting phase. These results are consistent with previous literature. They claimed that adding WO_3_ NPs (B) or (C), up to 25 mass%, improved the composites’ heat stability^45^. Additionally, as the filler loading increased, the DSC measurement for pure HDPE showed that the ability to induce HDPE nucleation was accompanied by a decline in the temperatures of fusion in all samples investigated.

## Conclusions

To clarify the impact of hexaferrite loading on their structural, mechanical, and thermal properties, BaSrCa/HDPE composites were successfully produced and thoroughly assessed. With a decrease in crystallite size from 59.31 to 50.38 nm and an increase in volume fraction from ~ 4.9% to ~ 19.3% as filler content increased from 5 to 20 wt%, XRD, and Rietveld refinement verified the gradual inclusion of the BaSrCa phase. The minor contraction of HDPE lattice properties indicates increased interfacial tension and limited polymer chain mobility at higher filler concentrations. Increasing the BaSrCa level significantly improved stiffness and strength, according to mechanical testing. At 20 wt% filler, Young’s modulus increased by over 30%, and yield stress increased from 16.48 MPa for pure HDPE to 25.37 MPa, indicating better load transfer between the ceramic phase and the polymer matrix. But as the strain at break dropped from 536% to less than 100%, especially at large filler loadings, this strengthening was accompanied by a noticeable decrease in ductility. Thermal analyses further demonstrated that the addition of BaSrCa nanoparticles influences the thermal stability and degradation behavior of HDPE. Thermogravimetric analysis showed an increase in the residual mass from 2.41% for pure HDPE to 10.51% at 20 wt% filler, indicating enhanced char formation and improved thermal resistance. The degradation temperatures exhibited slight variations due to the catalytic effects of iron-based oxides present in the nanofillers. Differential scanning calorimetry results revealed a gradual increase in melting temperatures with increasing filler loading, suggesting that BaSrCa nanoparticles act as heterogeneous nucleating agents that promote polymer crystallization and improve thermal stability. Improved thermal stability was also found by thermal analysis, as shown by increased residual mass and a delayed commencement of degradation. Overall, HDPE is efficiently reinforced by BaSrCa nanoferrite fillers, which increase strength, stiffness, and heat resistance at the price of elongation. These findings highlight the potential of HDPE/BaSrCa nanocomposites as an ideal filler content of 10–15 wt% for advanced lightweight structural materials and multifunctional applications, particularly in areas requiring enhanced mechanical performance and improved thermal resistance.

## Data Availability

The datasets used and/or analyzed during the current study are available from the corresponding author on reasonable request.
